# Petrous apex lesion following meningitis: management and discussion

**DOI:** 10.1093/jscr/rjab371

**Published:** 2021-09-23

**Authors:** Charles Elmaraghy, Ryan Bishop, Christine Barron, Oliver Adunka

**Affiliations:** Department of Otolaryngology-Head and Neck Surgery, The Ohio State College of Medicine, Columbus, OH, USA; Department of Pediatric Otolaryngology–Head and Neck Surgery, Nationwide Children’s Hospital, Columbus, OH, USA; Department of Otolaryngology-Head and Neck Surgery, The Ohio State College of Medicine, Columbus, OH, USA; Department of Otolaryngology–Head and Neck Surgery, Mount Sinai, New York, NY, USA; Department of Otolaryngology-Head and Neck Surgery, The Ohio State College of Medicine, Columbus, OH, USA; Department of Pediatric Otolaryngology–Head and Neck Surgery, Nationwide Children’s Hospital, Columbus, OH, USA

## Abstract

A 9-year-old male with history of mixed hearing loss presented with petrous apex lesion following episode of meningitis. Serial imaging revealed persistence of the lesion necessitating biopsy to rule out malignancy. Biopsy revealed inflammatory changes. The management of petrous apex lesions following meningitis can be conservative but repeat imaging is necessary to rule out progression and to rule out neoplastic process.

## INTRODUCTION

We describe a case of petrous apicitis following bacterial meningitis in a child presenting with profound sensorineural hearing loss and progressive, bony erosions on imaging that prompted surgical intervention. Petrous apicitis is commonly associated with lesions of the fifth and sixth cranial nerves. However, sensorineural hearing loss and facial nerve paralysis are rare but important presenting symptoms for the clinician to remain aware of. Although petrous apicitis is traditionally described as an acute process, it has the potential to take an indolent course. These features, coupled with its capacity for bony destructive changes, may mimic a neoplastic process.

## CASE

A 9-year-old male was admitted to the pediatric intensive care unit with group A beta-hemolytic streptococcal meningitis and right-sided otorrhea. He had a history of recurrent acute otitis media and tympanostomy tubes, the most recent of which were placed 1 month prior to admission. At baseline, the patient had symmetric mild to moderate sensorineural hearing loss secondary to STRC-related non-syndromic deafness.

On admission, audiogram revealed new onset profound, right-sided sensorineural hearing loss. On day two of admission, the patient developed right facial nerve weakness, House-Brackmann grade III. magnetic resonance imaging (MRI) taken on day three revealed non-coalescent mastoiditis. Management consisted of intravenous cefepime, metronidazole and vancomycin; ciprofloxacin-dexamethasone ear drops; and oral dexamethasone. He was discharged on Day 8 with a peripherally inserted central catheter (PICC) line and prescribed a 14-day total course of antibiotics, ciprofloxacin-dexamethasone ear drops and 9 days of oral steroids with a taper.

**Figure 1:**
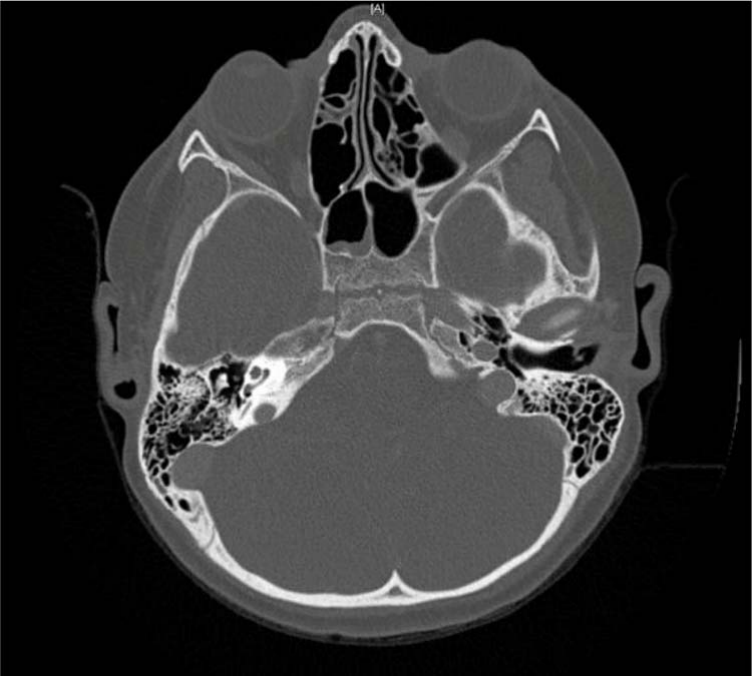
Axial CT taken 2 months after initial presentation demonstrates lucency of the right petrous apex representing partial pneumatization, as well as osseous erosion of the posterior cortex.

**Figure 2:**
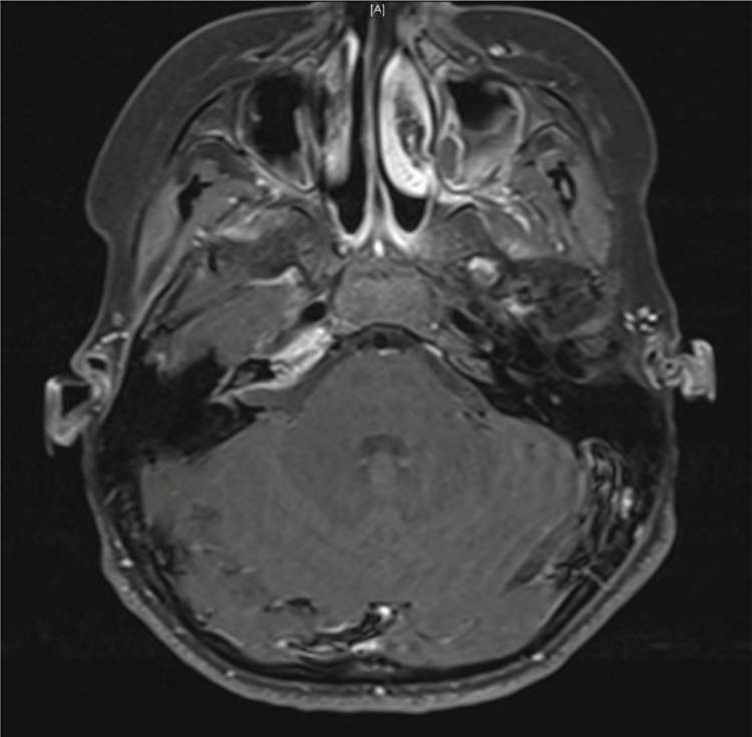
Axial contrast-enhanced T1-weighted image taken 3 months after admission shows enhancement within the right petrous apex and anterior aspect of the internal auditory canal.

Serial MRIs were conducted following discharge. MRI at 1 month showed a contrast-enhancing mass in the right internal auditory canal with enhancement of the right petrous apex and associated labyrinthitis. An MRI at 2 months showed persistence of the lesion, whereas computed tomography (CT) showed new osseous erosion and irregular lucency within the posterior cortex of the petrous apex (Figs 1 and 2). Given the patient’s hearing loss, persistence of the lesion and concern for neoplasm, surgical intervention was recommended to the family. Surgery was initially deferred, and serial imaging was recommended. The facial nerve palsy resolved and patient was followed closely as outpatient with family reluctant to pursue surgical intervention. However, repeat imaging at 4 months after the initial encounter demonstrated persistence of the lesion, and the family consented to surgery.

At surgery, the petrous apex was accessed using a translabyrinthine approach. Biopsies taken from the right vestibular nerve and the internal auditory canal showed no evidence of malignancy. Intraoperative findings included extensive scar tissue formation in internal auditory canal and labyrinthitis ossificans with a non-patent labyrinth. Gram stain, acid fast bacillus (AFB), fungal cultures and anaerobic cultures were negative. Clinically, the patient’s recovery was unremarkable.

## DISCUSSION

Petrous apicitis is suppurative inflammation of the petrous apex with parallel findings in the middle ear and mastoid [1]. Although the advent of modern antibiotics has rendered petrous apicitis a rare condition in children, it still occurs in ~2 per 100 000 with acute otitis media [2]. The petrous apex is pneumatized in 9–30% of individuals and often parallels mastoid aeration [1]. Most cases of acute petrous apicitis occur from extension of middle ear-mastoid infection into previously pneumatized petrous apex air cells. However, rare cases, such as with our patient, have been described in partially or non-pneumatized temporal bones. It has been theorized that these cases may represent either true osteomyelitis or spread of infection via diploeic veins of the petrous apex [3, 4].

Petrous apicitis is classically associated with Gradenigo’s syndrome, first described in 1907 as otorrhea, ipsilateral sixth nerve palsy and retroorbital pain [5]. This triad can be attributed to the close proximity of the petrous apex to several important anatomical structures. Along the superior surface of the petrous apex lies the trigeminal ganglion within Meckel cave, accounting for retroorbital pain secondary to trigeminal neuropathy. Likewise, the abducens nerve passes over the superior margin of the petrous apex before entering the Dorello canal, as it travels toward the cavernous sinus, leaving it susceptible to compression. Notably, the petrous apex also lies in close proximity to the facial and vestibulocochlear nerves as they enter the internal auditory canal on the posterior margin [1, 6].

Profound sensorineural hearing loss, as seen in our patient, is rarely described in older case reports as a prominent feature of petrous apicitis [5]. In the pediatric population, hearing loss secondary to petrous apex lesions is more frequently associated in the literature with cholesterol granuloma, meningiomas or other neoplasms, and petrous carotid artery aneurysms [1, 4, 6, 9]. Despite this, the same case series of 44 adults found the incidence of facial and vestibulocochlear nerve lesions together were almost twice as common as those involving the abducens nerve [5]. Whether this holds true in the pediatric population remains to be examined closely in the literature.

Finally, although petrous apicitis is most commonly described in its acute form, it has the potential to manifest as a chronic, indolent process that mirrors the histopathologic changes seen in chronic middle ear disease [3, 7, 8, 10–12]. Chronic fibrous proliferation leads to bony erosion and permanent thickening of the petrous mucosal membranes, resulting in a central cystic lesion that makes the disease difficult to treat with antibiotics [10]. As seen in our patient, imaging may reveal poorly pneumatized cells, enhancement of the overlying dura and bony erosion that mimics malignancy, metastasis, or cholesterol granuloma [6, 13]. Infection can persist for months with varying degrees of activity, resulting in either spontaneous resolution or progression to complications such as meningitis and labyrinthitis [10, 11].

## CONCLUSION

Sensorineural hearing loss and facial nerve paralysis are important presenting symptoms of petrous apicitis. Furthermore, petrous apicitis has the potential to mimic a neoplastic process. These characteristics should be considered in the differential diagnosis of pediatric petrous apex lesions when the clinical presentation is appropriate.
